# Supplementary material for: Structural organization of HBV pgRNA genome driven by phase separation in capsid confinement
Source: Nat Commun. 2026 Feb 19;17:2940. doi: 10.1038/s41467-026-69689-2 (PMC13031878; doi:10.1038/s41467-026-69689-2)
Supplement: Supplementary file 1 — Supplementary Information [file 41467_2026_69689_MOESM1_ESM.pdf]

Supplementary Material for Structural organization of HBV pgRNA genome driven by phase separation in capsid confinement

Yunqiang Bian,[†] Hai Pan,[†] Jiaqi Mao,[†,‡] Yixin He,[¶,†] Yanwei Wang,[¶,‡] Yi Cao,[¶,†] Wenfei Li,[*,¶,†,§] and Wei Wang[¶]

†*Wenzhou Key Laboratory of Biophysics, Wenzhou Institute, University of Chinese Academy of Sciences, Wenzhou, Zhejiang 325000, China*

‡*Department of Physics, Wenzhou University, Wenzhou 325035, China*

¶*Department of Physics, National Laboratory of Solid State Microstructure, Nanjing University, Nanjing 210093, China*

§*Jiangsu Key Laboratory for Cardiovascular Information and Health Engineering Medicine, Nanjing Drum Tower Hospital, Medical School, Nanjing University, Nanjing 210093, P. R. China.*

E-mail: wfli@nju.edu.cn

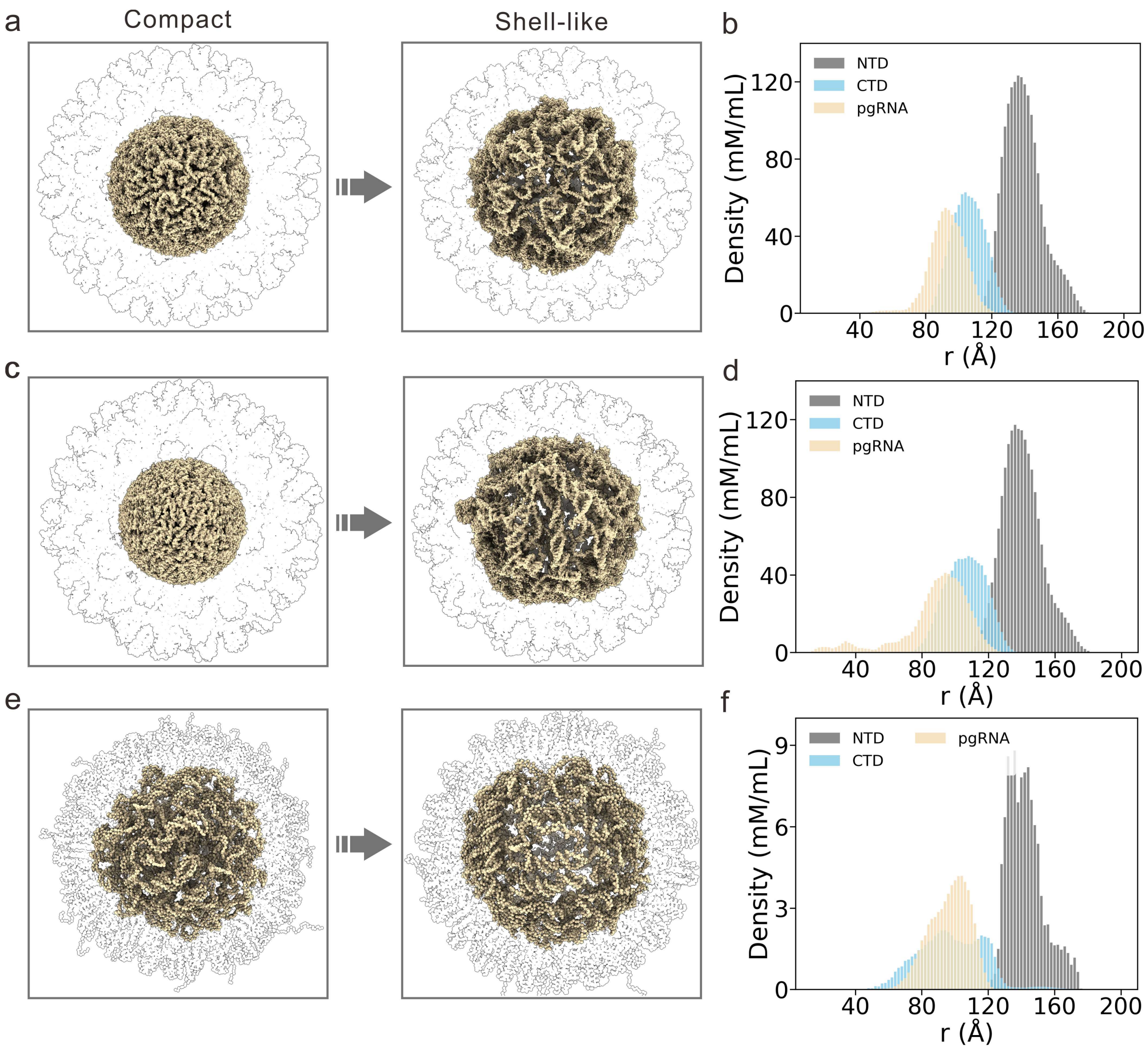

Supplementary Figure 1. Conformations of pgRNA from the MD simulations with different initial structures. (a,b) The starting and final configurations (a), and the corresponding radial distribution functions of NTD (gray), CTD (blue), and pgRNA (wheat) in the final configuration (b), from the all-atom MD simulation initialed from an unstructured pgRNA. (c,d) Same as (a,b), but for the all-atom MD simulation initiated from a pgRNA structure predicted by AlphaFold3. (e,f) Same as (a,b) but for the CG MD simulation initiated from a pgRNA structure predicted by AlphaFold3.

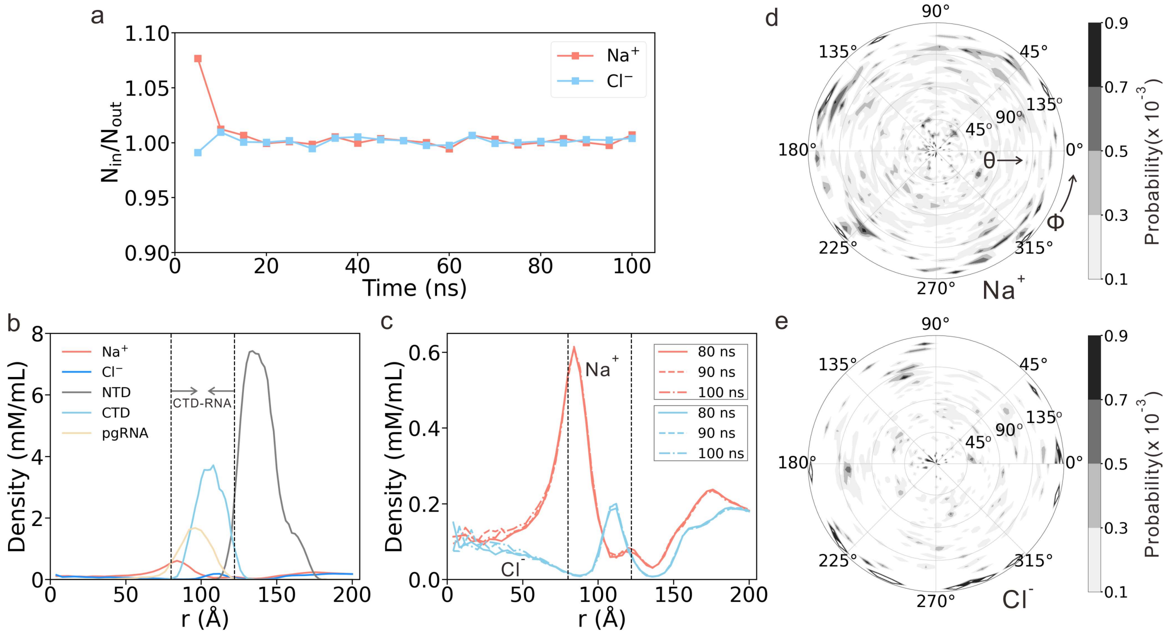

Supplementary Figure 2. Distribution of mobile ions estimated from the all-atom MD simulations. (a) Ratio of the cumulative number of Na^+ (red) and Cl^- (blue) ions flowing into and out of the capsid over successive 5 ns intervals. The ratio converges to ∼1.0, indicating equilibrium of ion motions across the capsid. (b) Radial distribution functions of Na^+ (red) and Cl^- (blue) relative to the capsid center. For comparison, the distribution of NTD (gray), CTD (light blue), and pgRNA (wheat) were also shown. The phosphate beads were used in calculating the distribution of pgRNA. (c) Radial distribution functions of Na^+ (red) and Cl^- (blue) relative to the capsid center calculated at three different simulation times (80ns, 90ns, and 100ns). The distributions at different times are comparable, suggesting again the convergence of the sampling for ion motions. (d,e) Representative two-dimensional density distributions of Na^+ (a) and Cl^- (b) plotted along spherical coordinates (θ, ϕ). The density distributions in (d,e) were calculated for the region indicated by two vertical lines in (b,c).

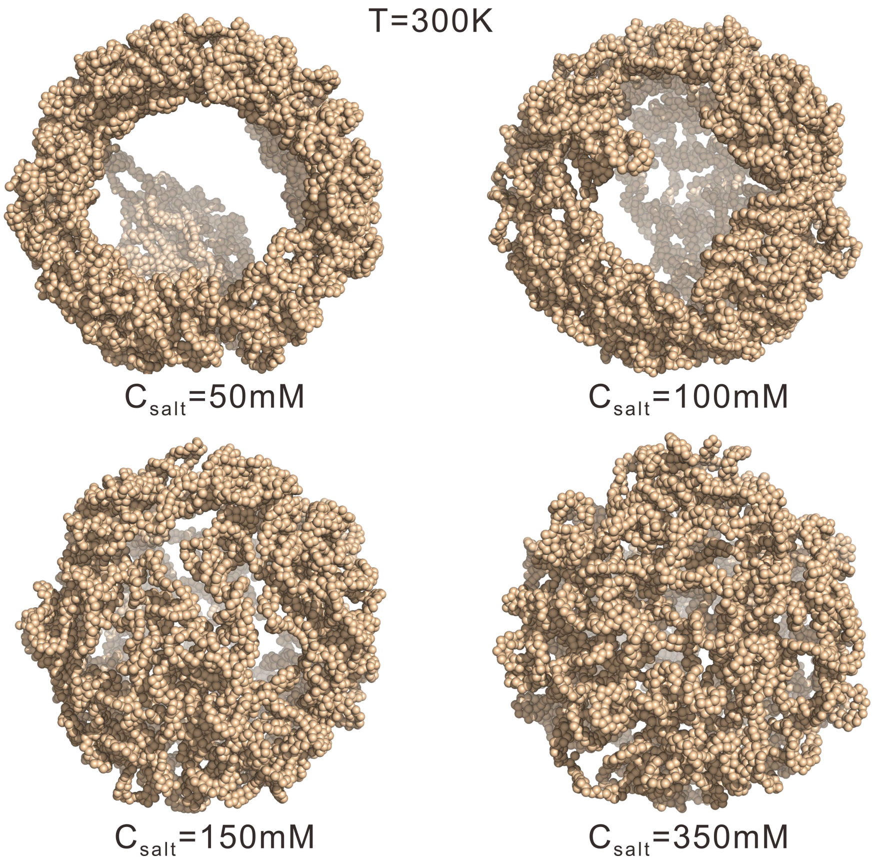

Supplementary Figure 3. Representative conformations sampled by CG simulations under varying salt concentrations at 300 K.

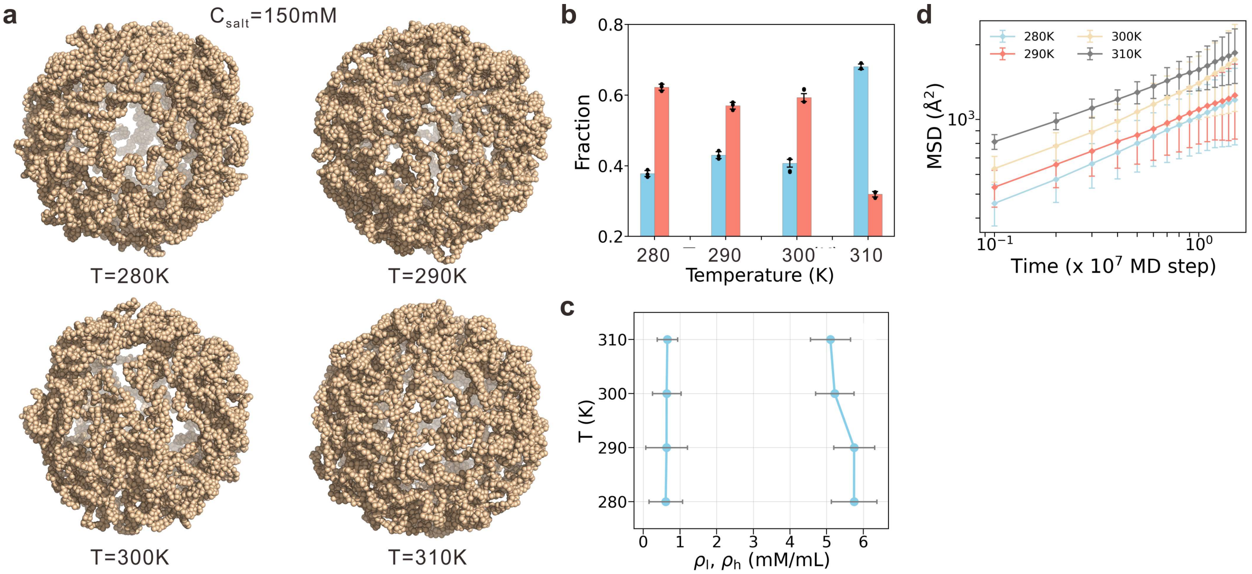

Supplementary Figure 4. Modulation of phase separation by temperature. (a) Representative conformations sampled by CG simulations under varying temperatures at 150 mM salt. (b) Fraction of free (blue) and condensed (red) nucleotides as a function of temperatures. Data are presented as mean +/- standard deviation (SD) from 3 independent simulations. (c) Phase diagram as a function of temperature (T), mapped via the mean densities of the low- (ρ_l) and high-density (ρ_h) regions. Data are presented as mean +/- SD from 1000 independent simulation snapshots under each temperature. (d) MSD of pgRNA, averaged over different 100-nt segments of the full-length pgRNA, under different temperatures. Data are presented as mean +/- SD derived from 32 RNA segments of 100-nt in length.

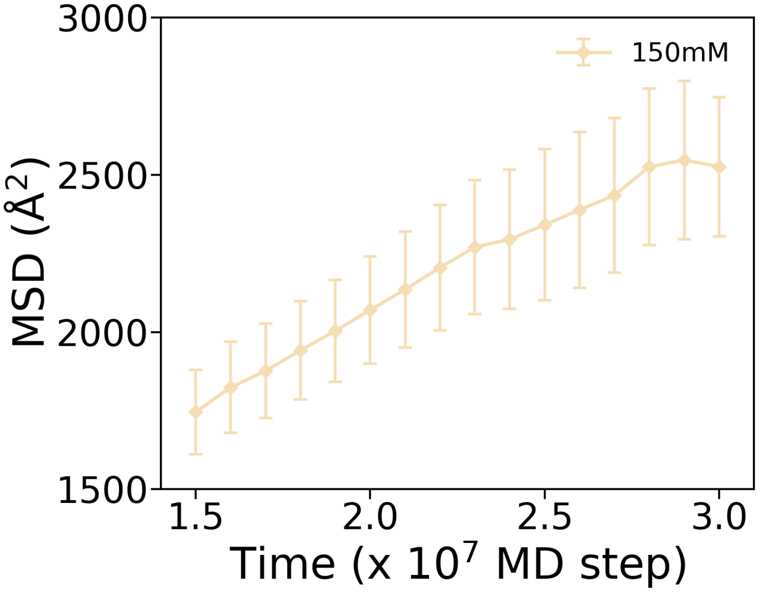

Supplementary Figure 5. Mean-squared displacement (MSD) of pgRNA, averaged over different 100-nt segments of the full-length pgRNA from CG simulations at temperature of 300K and salt concentration of 150 mM. The slope of the MSD plot gets smaller at longer lag-time because of the capsid confinement. Data are presented as mean +/- SD derived from 32 RNA segments of 100-nt in length.

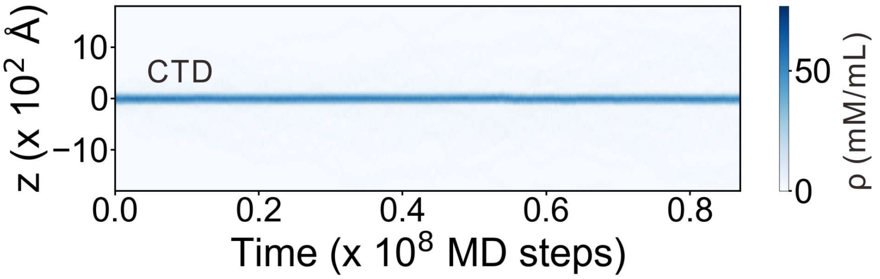

Supplementary Figure 6. Z-axis density profiles of CTD peptides in the slab simulation trajectory.

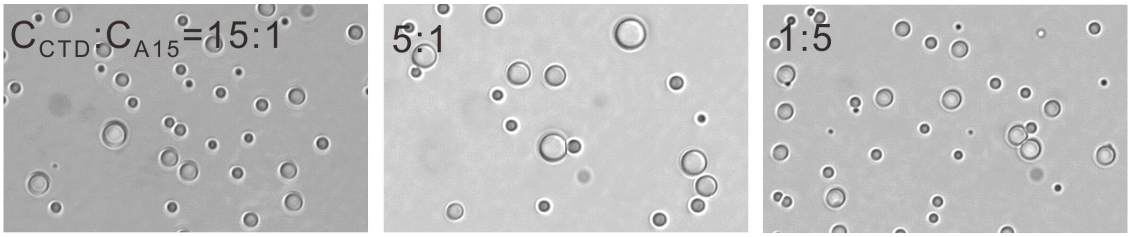

Supplementary Figure 7. Bright field droplet assays with varying CTD:RNA molar mixing ratios.

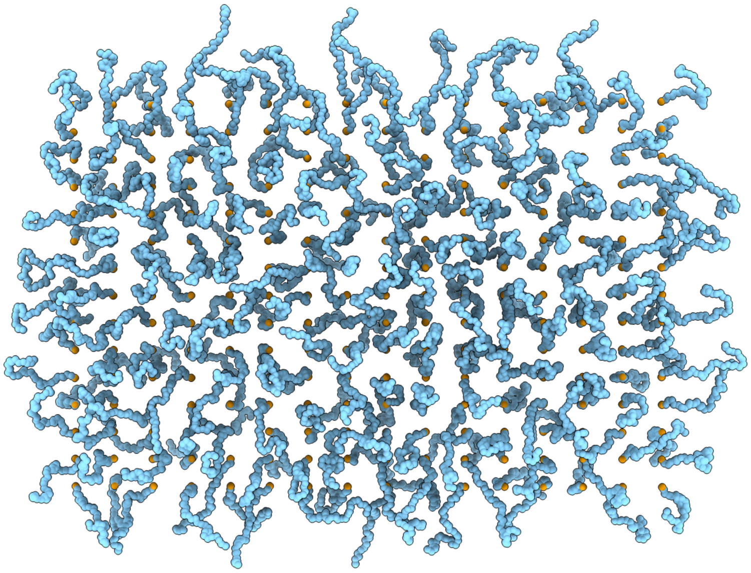

Supplementary Figure 8. Representative simulation snapshot of CTD-layer only system from CG simulations at 300 K and 150 mM salt. Phase separation was not observed for this system.

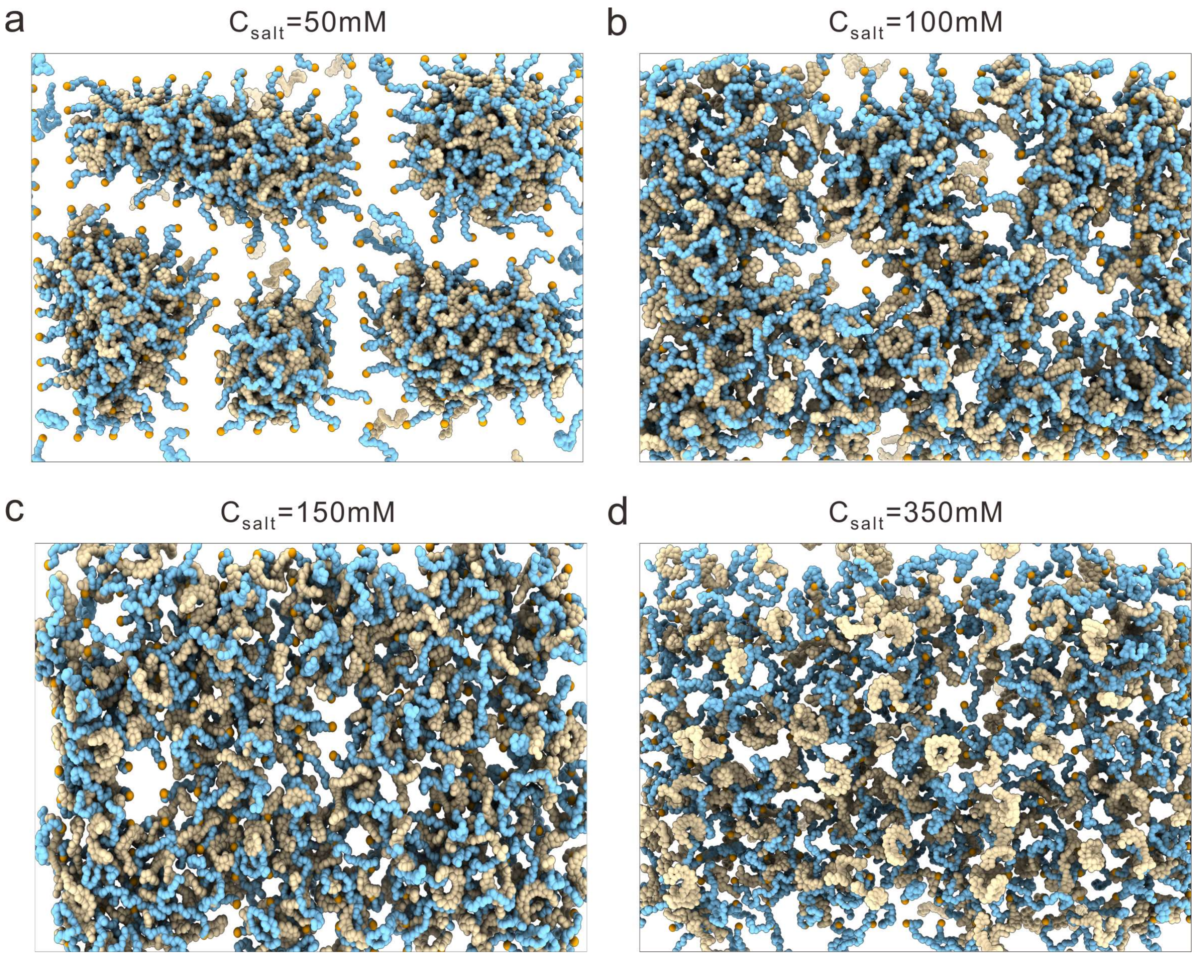

Supplementary Figure 9. Representative simulation snapshots from CG simulations of the two-dimensional system at a salt concentration of (a) 50mM, (b) 100mM, (c) 150 mM, and (d) 350mM for A_{15} RNA molecules along the CTD layer. The CTD and RNA chains are colored blue and wheat, respectively.

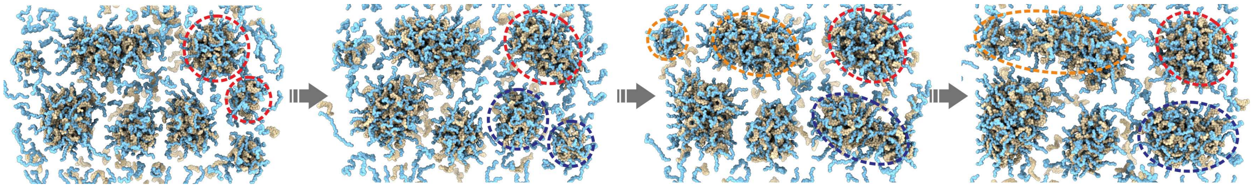

Supplementary Figure 10. Fusion events captured in CG simulations of A_{15} RNA molecules along the two-dimensional CTD layer at the salt concentration of 50 mM. The CTD and RNA chains are colored blue and wheat, respectively.

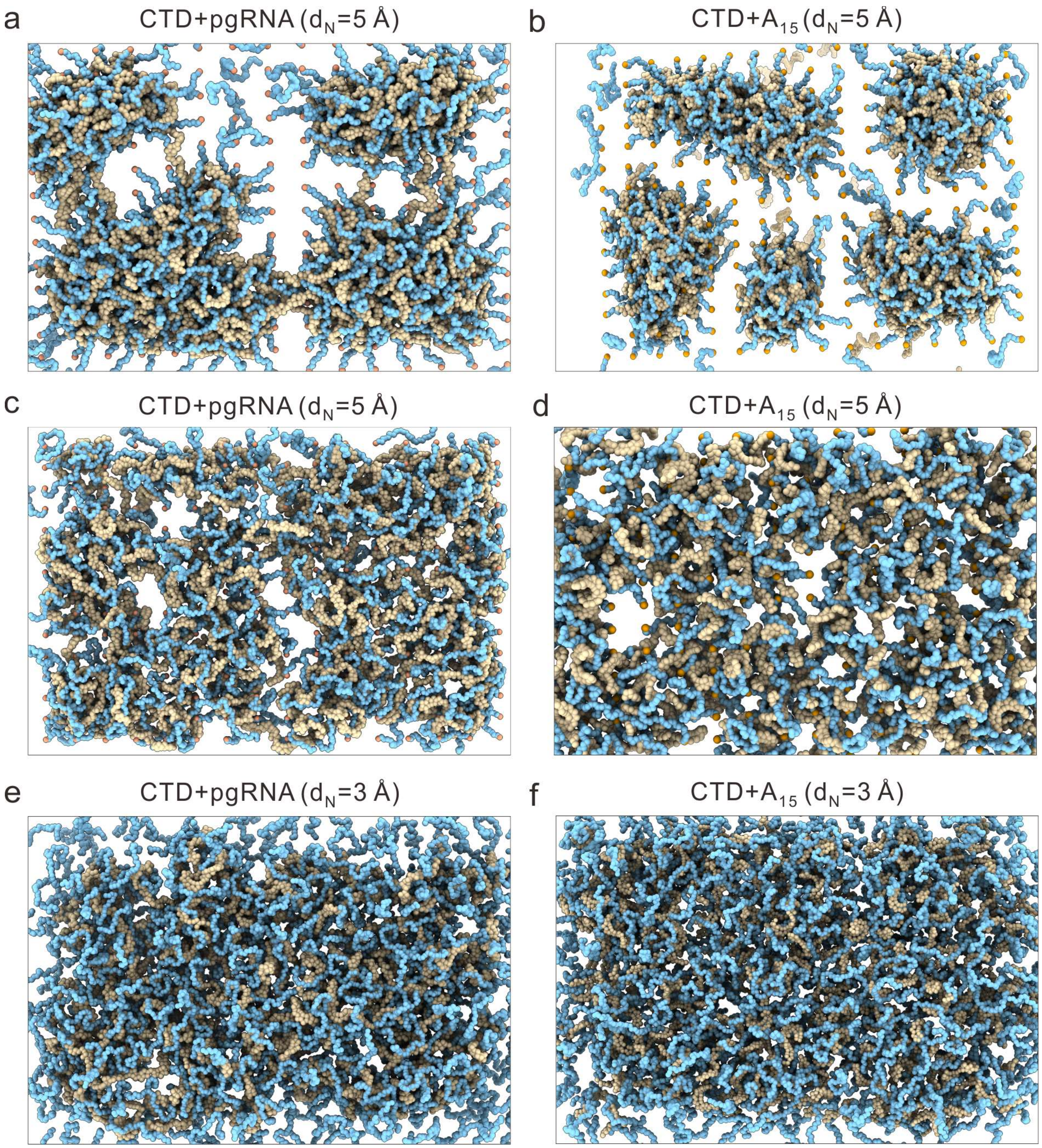

Supplementary Figure 11. Effect of CTD density on the CTD-RNA condensation in the two-dimensional system. (a,b) Representative snapshots for the CG simulations with the addition of pgRNA (a) and A_{15} RNA (b) to the two-dimensional system with the native CTD density at the salt concentration of 50mM. (c,d) Same as (a,b) but at the salt concentration of 150mM. (e,f) Same as (a,b) but with increased CTD density at the salt concentration of 150mM. d_N refers to the distance between the N-terminal anchoring beads of neighboring CTDs. The CTD and RNA chains are colored blue and wheat, respectively.

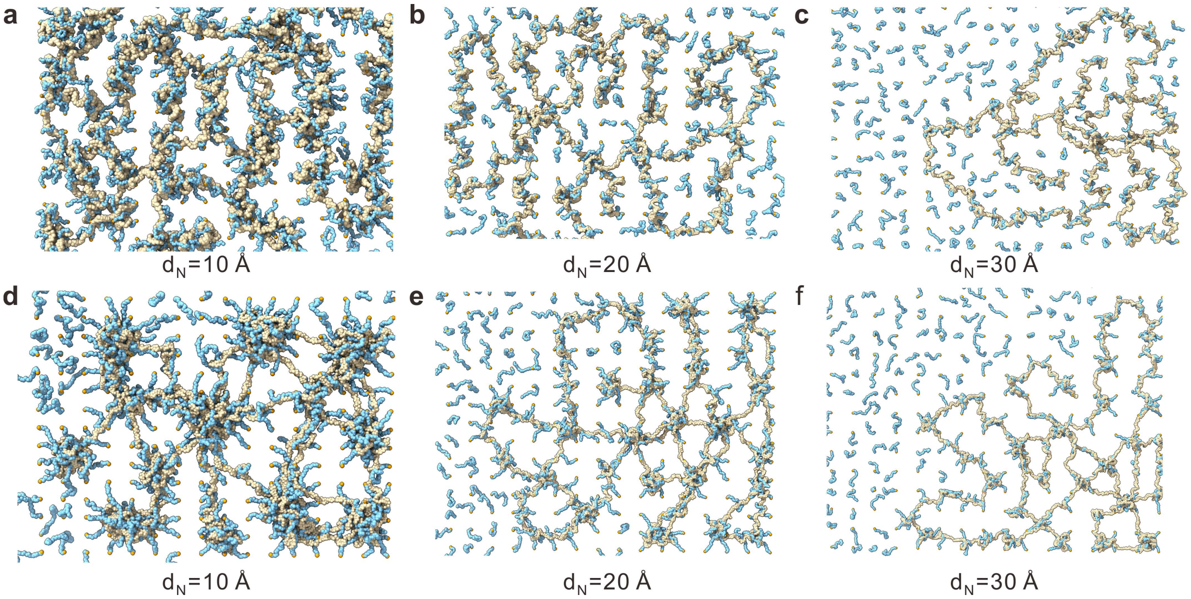

Supplementary Figure 12. Modulation of phase separation through CTD spacing variation from CG simulations at the salt concentration of 150 mM (**a-c**) and 50 mM (**d-f**). The CTD and RNA chains are colored blue and wheat, respectively.

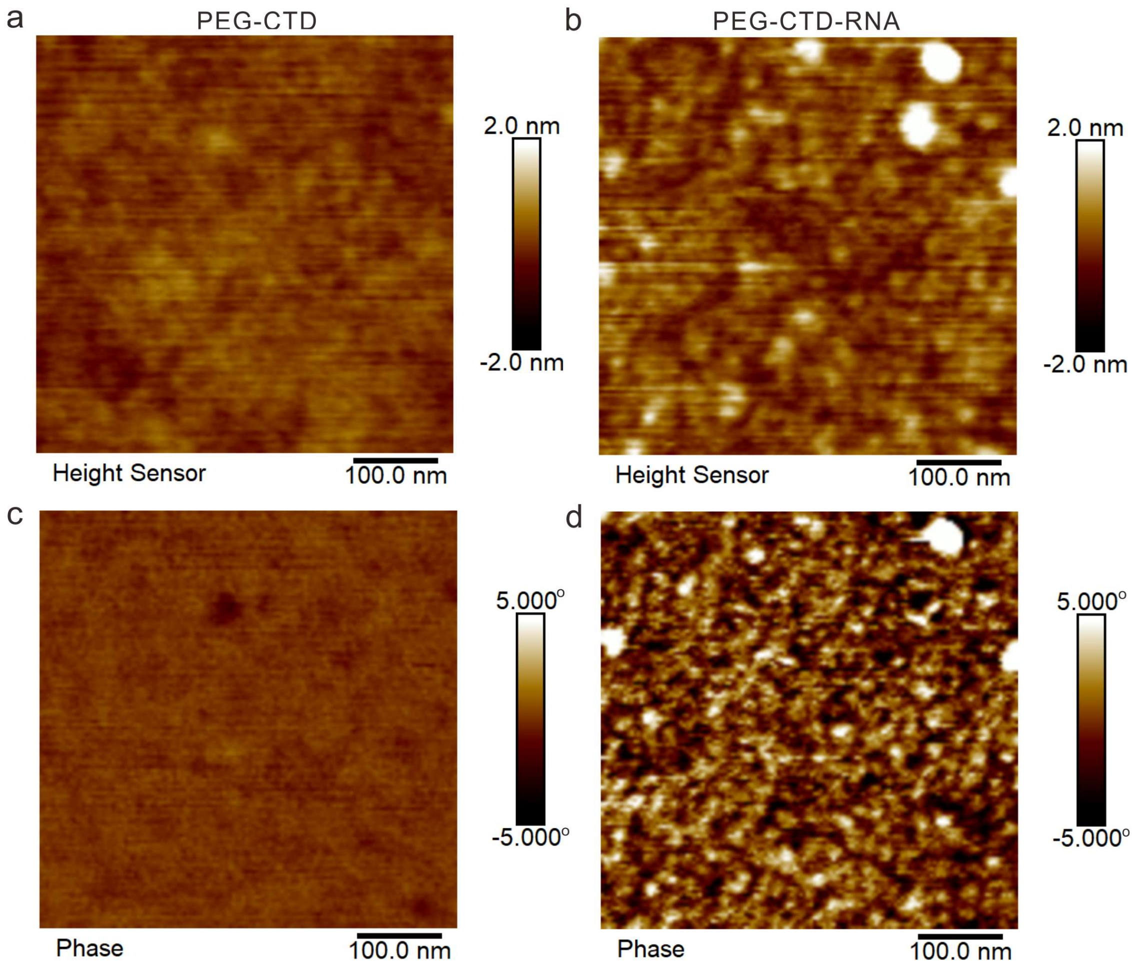

Supplementary Figure 13. AFM characterization of RNA induced mophological transition of PEG-CTD layer. (a,b) AFM tapping mode height images of PEG-CTD layer before (a) and after (b) the addition of A_{15} RNA. (c,d) AFM phase images of PEG-CTD layer before (c) and after (d) the addition of A_{15} RNA.

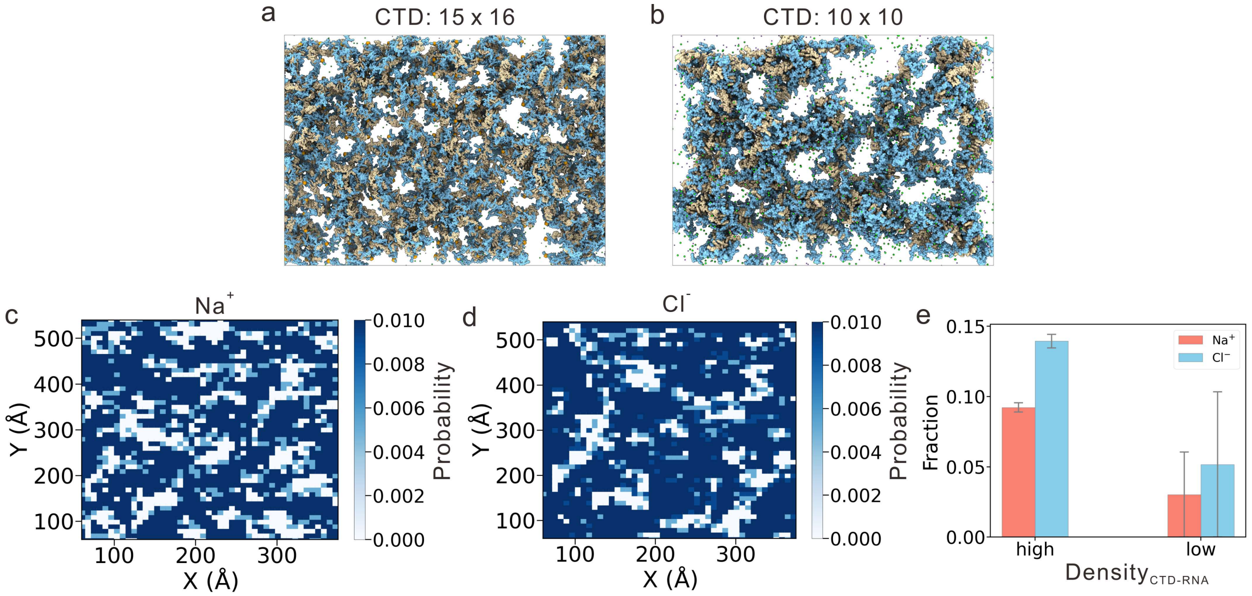

Supplementary Figure 14. All-atom MD simulations of phase separation of the two-dimensional CTD-RNA system. (a) Representative snapshot of all-atom MD simulation for the two-dimensional system containing 15×16 array of CTD chains with the simulation length of 100 ns. The CTD and RNA chains are colored blue and wheat, respectively. (b) Representative snapshot of all-atom MD simulation for the two-dimensional system containing 10×10 array of CTD chains with the simulation length of 200 ns. The same color scheme in panel (a) was applied. (c,d) Distribution of Na^+ (c) and Cl^- (d) across the two-dimensional layer for the two-dimensional system containing 15×16 array of CTD chains. (e) Fraction of Na^+ (red) and Cl^- (blue) within the high- and low-density regions of CTD-RNA clusters. Data are presented as mean +/- SD derived from 1000 independent simulation snapshots.

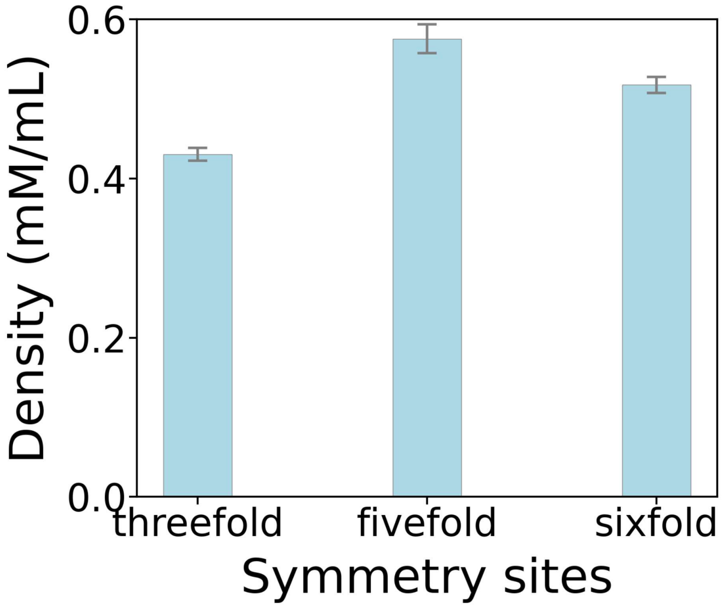

Supplementary Figure 15. Density of CG beads around different symmetry sites estimated from CG simulations. Data are presented as mean +/- SD derived from 1000 independent simulation snapshots.

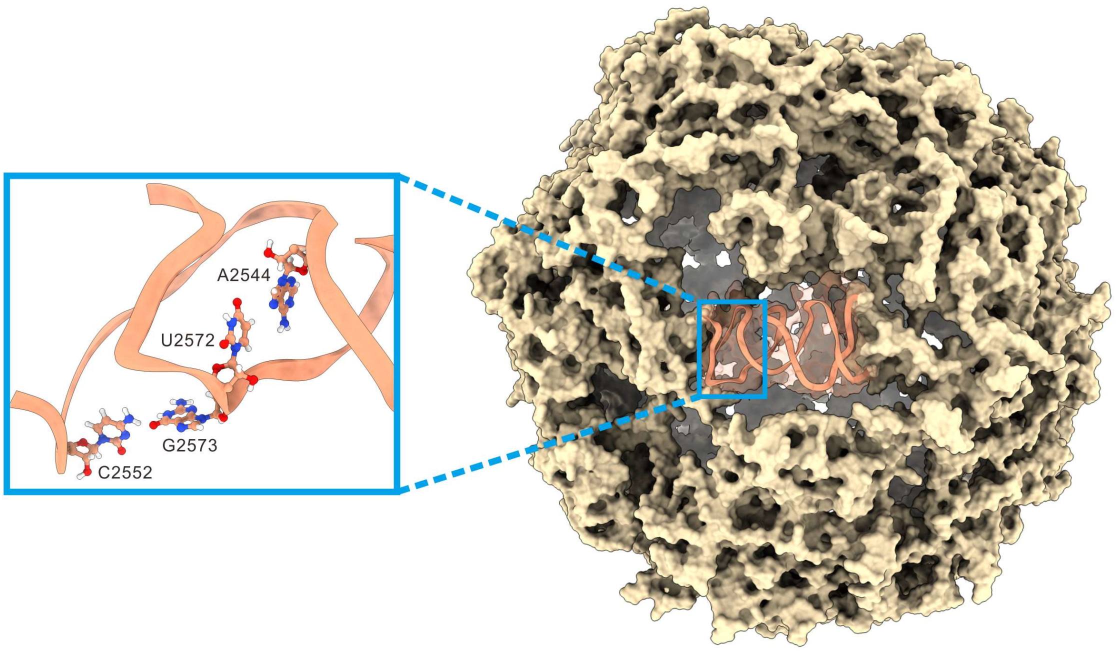

Supplementary Figure 16. Representative all-atom simulation snapshot illustrating the formation of dsRNA structures of pgRNA encapsuled in HBV capsid.

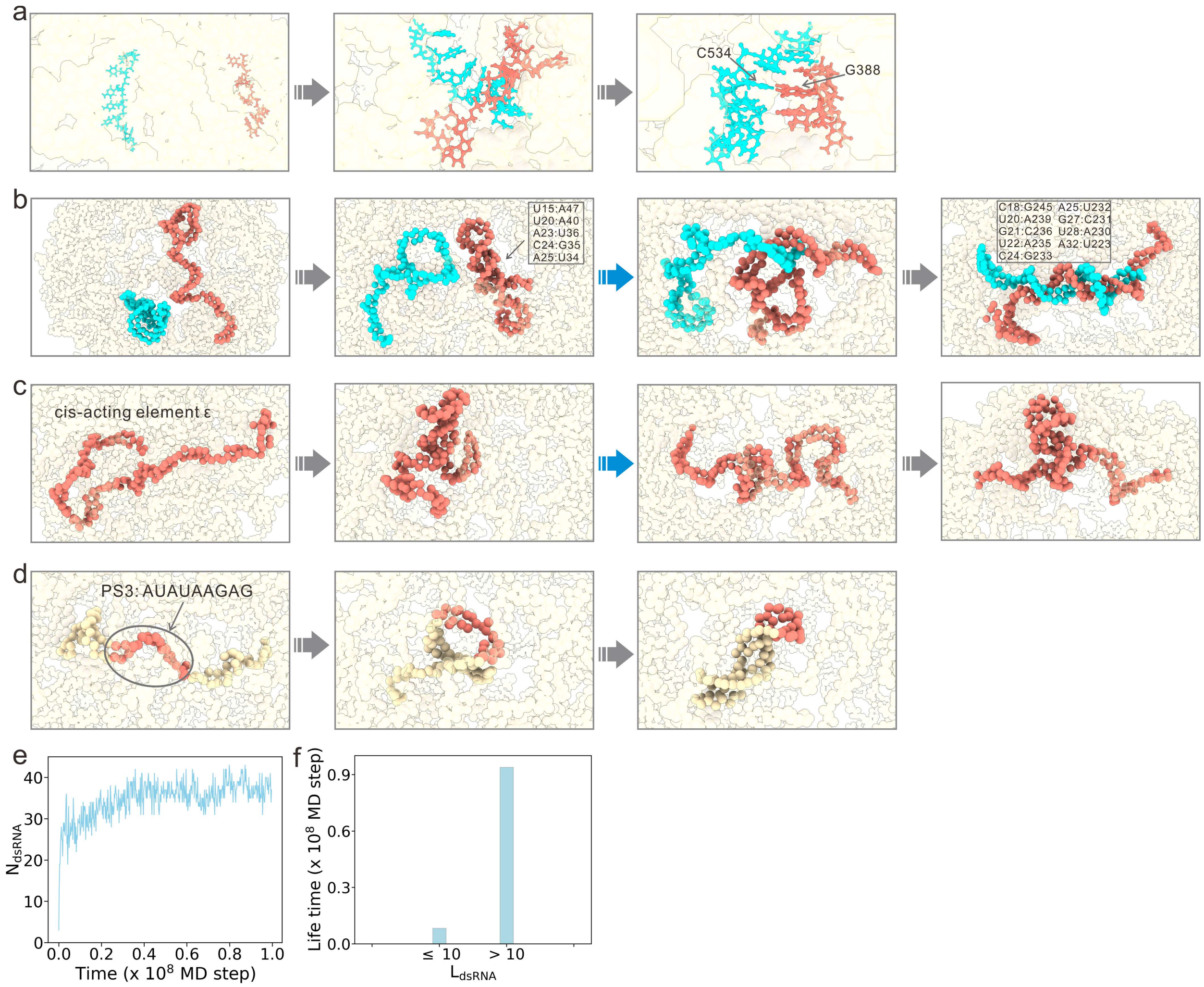

Supplementary Figure 17. Dynamics of base pairs in MD simulations. (a, b) Snapshots of representative base-pairing events from all-atom (a) and coarse-grained (CG) simulations (b). The two RNA segments forming base pairs are highlighted in red and blue, respectively. (c) Representative folding and unfolding transitions of the cis-acting element ε observed in CG simulations. (d) Representative folding event of packing site, PS3. (e) Time evolution of the number of dsRNA segments from CG simulations. (f) Lifetimes of dsRNA segments with lengths (L_{dsRNA}) $\leq$ 10 and >10. The lifetimes of dsRNA were estimated using a maximum-likelihood method.

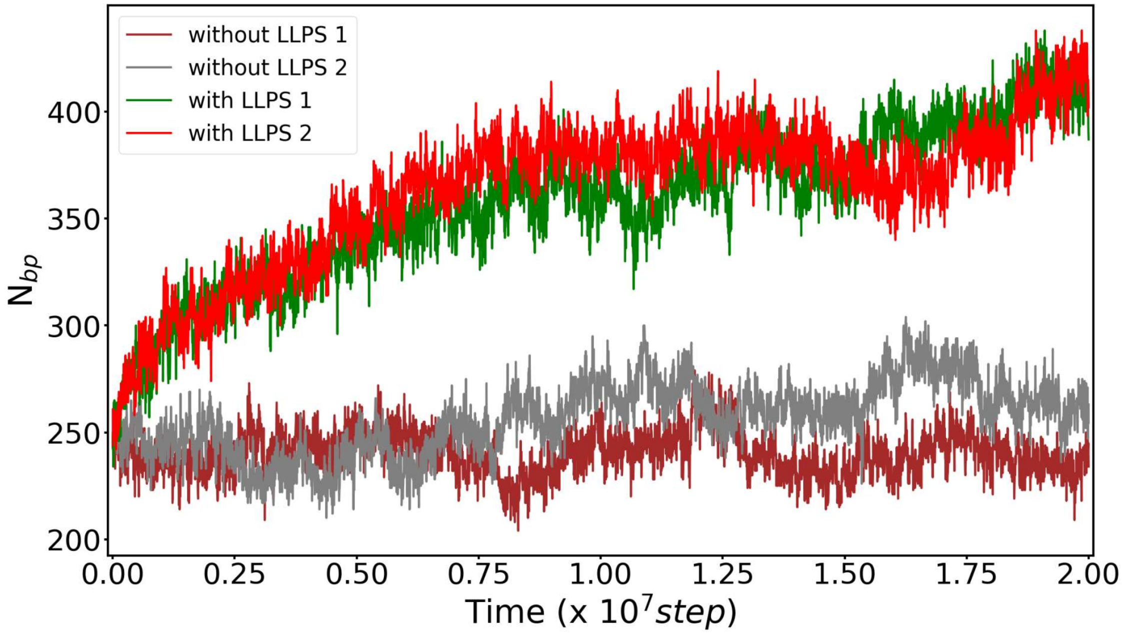

Supplementary Figure 18. Temporal evolution of the number of base pairs (N_{bp}) from CG simulations of the pgRNA-containing HBV capsid under conditions with (red and green) and without (gray and brown) the occurrence of phase separation, starting from a pgRNA conformation with predicted secondary structures. Two independent simulations were conducted for each case.

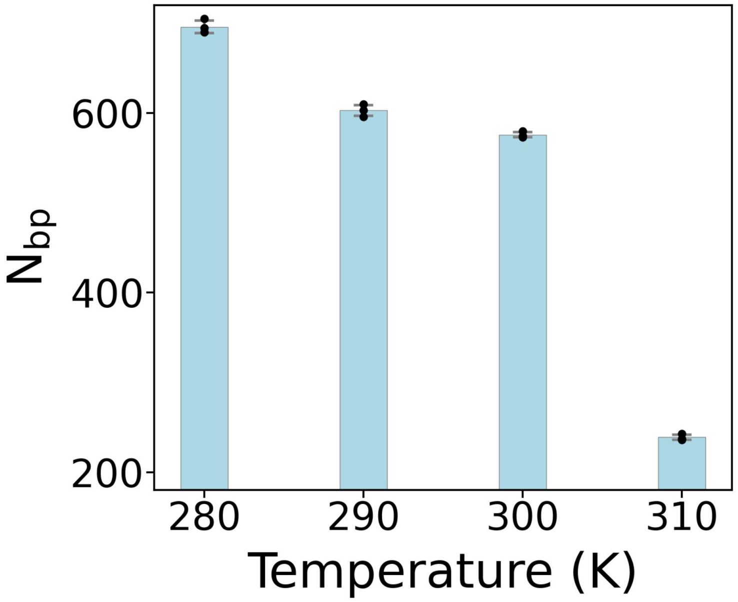

Supplementary Figure 19. Mean N_{bp} as a function of temperature from CG simulations of the pgRNA-containing HBV capsid at the salt concentration of 150mM. Data are presented as mean +/- SD derived from 3 independent simulations.

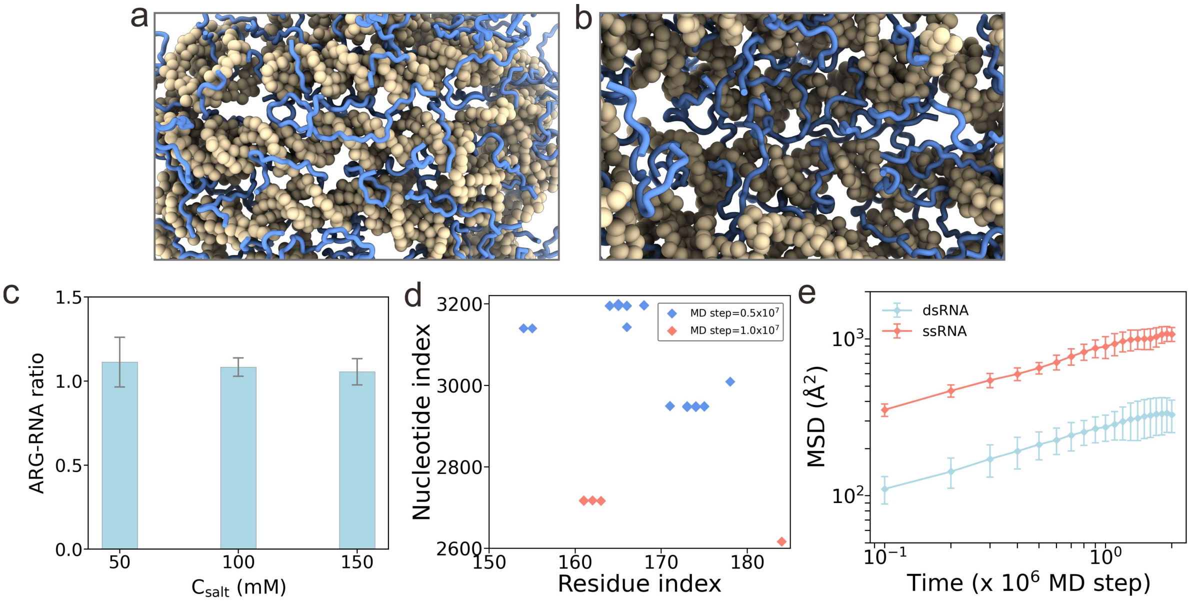

Supplementary Figure 20. Interaction modes between CTD and dsRNA from CG simulations of the pgRNA-containing HBV capsid. (a,b) A representative conformation showing the interactions between CTD (blue) and dsRNA (wheat) as viewed from the exterior (a) and interior (b) of HBV capsid, respectively. (c) Ratio of the numbers of arginine in CTDs and the nucleotides in RNA within high-density regions under different salt concentrations where phase separation is prominent. Data are presented as mean +/- SD derived from 1000 independent simulation snapshots. (d) The contact map between a randomly selected CTD segment and a specified pgRNA segment at two different simulation snapshots ($0.5x10^7$, blue; $1.0x10^7$, red). The large change of the contact maps suggests the dynamic nature of the base pairs. (e) MSD of dsRNA (blue) and ssRNA (red) within a short lag time. Data are presented as mean +/- SD derived from 20 ds/ssRNA segments.

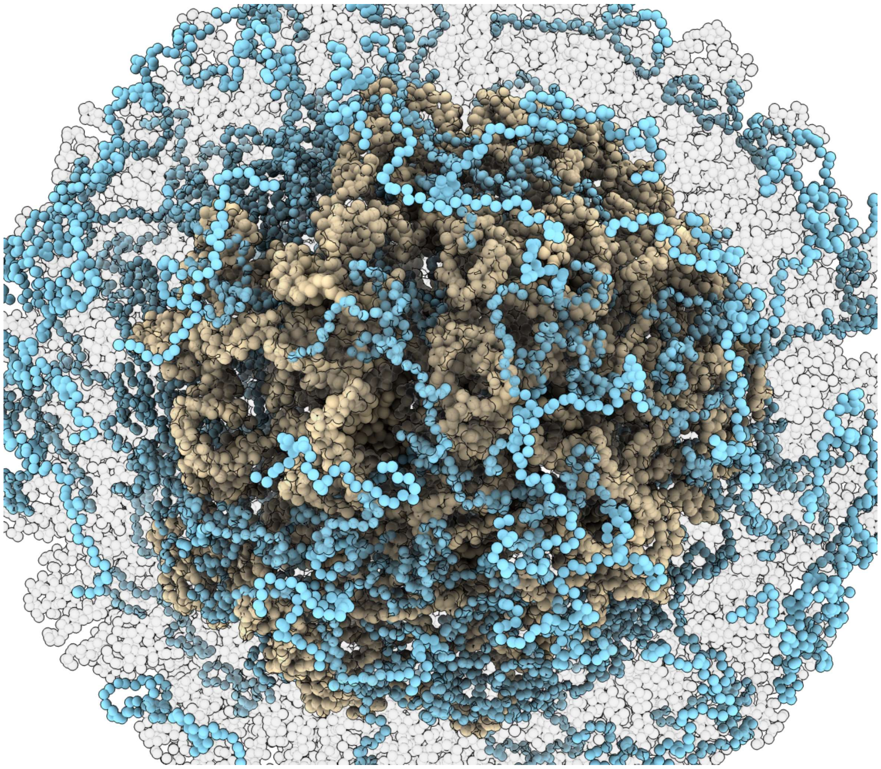

Supplementary Figure 21. Representative CG simulation snapshot illustrating the conformation of CTD-free system. The NTD, CTD and pgRNA are colored gray, blue and wheat, respectively.

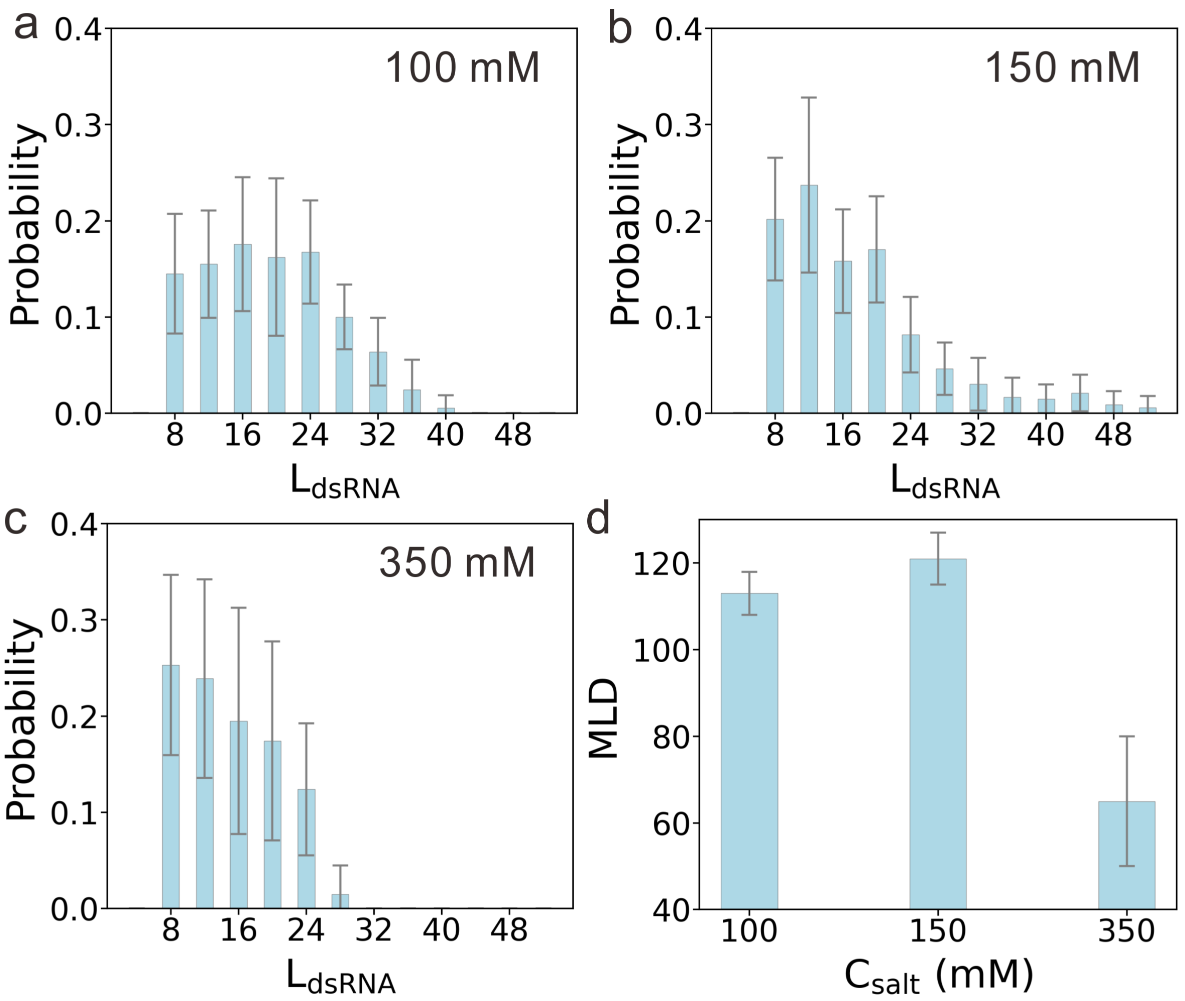

Supplementary Figure 22. Structural characterization of dsRNA. (a-c) Length distributions of dsRNA segments (L_{dsRNA}) from CG simulations of the pgRNA-containing HBV capsid under varying salt concentrations. The average dsRNA length decreases with increasing salt concentration. (d) Maximum ladder distance (MLD) of pgRNA as a function of salt concentration. The MLD becomes much smaller at the salt concentration of 350 mM, likely reflecting the weakened phase separation. Data in (a-d) are presented as mean +/- SD derived from 1000 independent simulation snapshots under each salt concentration.

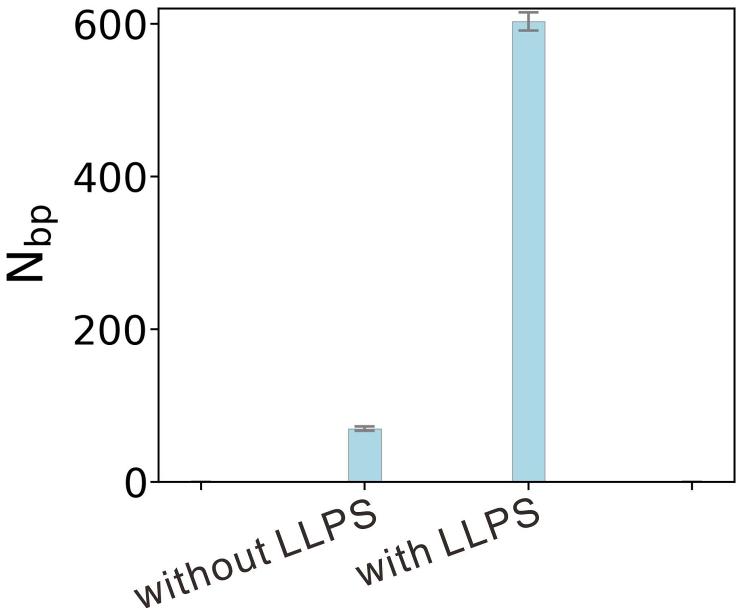

Supplementary Figure 23. Number of base pairs (N_{bp}) formed by long-range nucleotides (with sequence separation greater than 100) from CG simulations of the pgRNA-containing HBV capsid under conditions with and without the occurrence of LLPS. Data are presented as mean +/- SD derived from 1000 independent simulation snapshots under each condition.

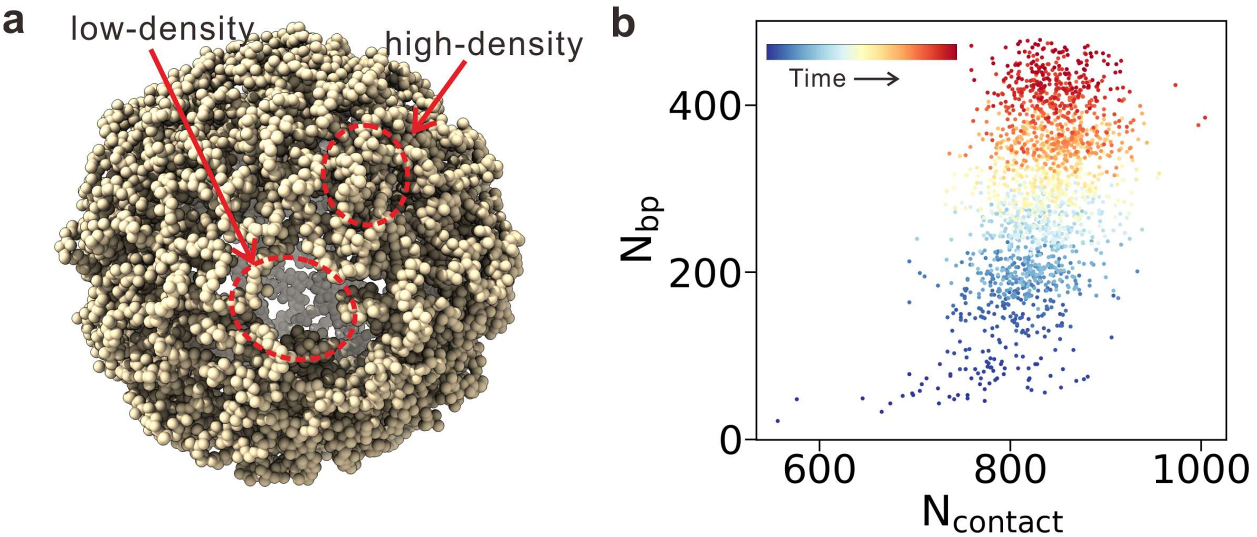

Supplementary Figure 24. CG simulation of pgRNA-filled HBV capsid in the presence of polymerase. **a** Representative conformation condensates depicting the coexisting low- and high-density regions. **b** Correlation between the number of CTD–pgRNA contacts ($N_{contact}$) and the number of base pairs (N_{bp}), along the time course of a representative MD trajectory.

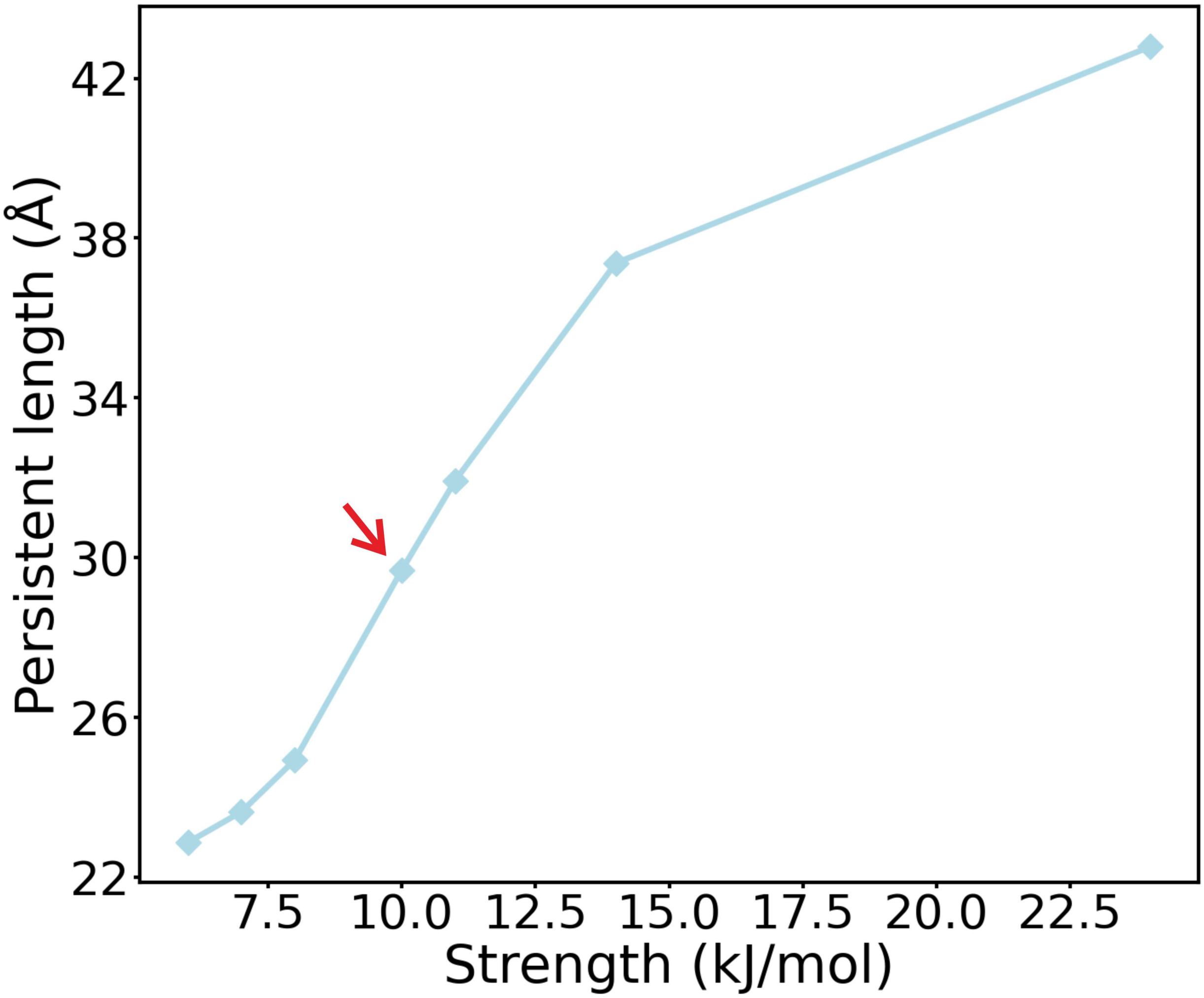

Supplementary Figure 25. Estimation of persistent length for sequence T40 through variation in dihedral energy strength in CG simulations. The red arrow signifies the value aligning with the persistent length of U40.
