# Supplementary material for: Structural organization of HBV pgRNA genome driven by phase separation in capsid confinement
Source: Nat Commun. 2026 Feb 19;17:2940. doi: 10.1038/s41467-026-69689-2 (PMC13031878; doi:10.1038/s41467-026-69689-2)
Supplement: Supplementary file 2 — Description Of Additional Supplementary File [file 41467_2026_69689_MOESM2_ESM.pdf]

Description of Additional supplementary files

Title: Supplementary Movie 1
Description: An all-atom simulation showing the coexisting low- and high-density regions within pgRNA structure.

Title: Supplementary Movie 2
Description: An all-atom simulation showing the formation of the shell-like pgRNA structure starting from an unstructured conformation.

Title: Supplementary Movie 3
Description: An all-atom simulation showing the formation of the shell-like pgRNA structure starting from a structured conformation.

Title: Supplementary Movie 4
Description: A coarse-grained simulation showing the formation of the shell-like structure of pgRNA starting from a structured conformation.

Title: Supplementary Movie 5
Description: A coarse-grained simulation showing the high dynamics of pgRNA within the capsid.

Title: Supplementary Movie 6
Description: A coarse-grained slab simulation showing the coexistence of dilute and dense phases.

Title: Supplementary Movie 7
Description: A fusion event captured in experiments.

Title: Supplementary Movie 8
Description: A coarse-grained simulation of pgRNA along two dimensional CTD membrane.

Title: Supplementary Movie 9
Description: Typical fusion events in coarse-grained simulation of A15 RNA molecules along two dimensional CTD membrane.

Title: Supplementary Movie 10:
Description: Formation of base pair observed in an all-atom simulation.

Title: Supplementary Movie 11
Description: Formation and unwinding of dsRNA observed in a coarse-grained simulation.

Title: Supplementary Movie 12

Description: Structural dynamics of the cis-acting regulatory element ε in a coarse-grained simulation.

Title: Supplementary Movie 13

Description: Structural dynamics of a packing site (PS3) in a coarse-grained simulation.
