# Supplementary material for: Structural organization of HBV pgRNA genome driven by phase separation in capsid confinement
Source: Nat Commun. 2026 Feb 19;17:2940. doi: 10.1038/s41467-026-69689-2 (PMC13031878; doi:10.1038/s41467-026-69689-2)
Supplement: Supplementary file 16 — Reporting summary [file 41467_2026_69689_MOESM16_ESM.pdf]

nature portfolio

Corresponding author(s): Wenfei Li

Last updated by author(s): 2026-1-19

Reporting Summary

Nature Portfolio wishes to improve the reproducibility of the work that we publish. This form provides structure for consistency and transparency in reporting. For further information on Nature Portfolio policies, see our Editorial Policies and the Editorial Policy Checklist.

Statistics

For all statistical analyses, confirm that the following items are present in the figure legend, table legend, main text, or Methods section.

n/a	Confirmed	
☐	☒	The exact sample size (*n*) for each experimental group/condition, given as a discrete number and unit of measurement
☐	☒	A statement on whether measurements were taken from distinct samples or whether the same sample was measured repeatedly
☒	☐	The statistical test(s) used AND whether they are one- or two-sided<br>*Only common tests should be described solely by name; describe more complex techniques in the Methods section.*
☒	☐	A description of all covariates tested
☒	☐	A description of any assumptions or corrections, such as tests of normality and adjustment for multiple comparisons
☐	☒	A full description of the statistical parameters including central tendency (e.g. means) or other basic estimates (e.g. regression coefficient) AND variation (e.g. standard deviation) or associated estimates of uncertainty (e.g. confidence intervals)
☒	☐	For null hypothesis testing, the test statistic (e.g. *F*, *t*, *r*) with confidence intervals, effect sizes, degrees of freedom and *P* value noted<br>*Give P values as exact values whenever suitable.*
☒	☐	For Bayesian analysis, information on the choice of priors and Markov chain Monte Carlo settings
☒	☐	For hierarchical and complex designs, identification of the appropriate level for tests and full reporting of outcomes
☒	☐	Estimates of effect sizes (e.g. Cohen's *d*, Pearson's *r*), indicating how they were calculated

Our web collection on statistics for biologists contains articles on many of the points above.

Software and code

Policy information about availability of computer code

Data collection	1. All-atom MD simulations were performed using the Gromacs 2020.4 (https://www.gromacs.org).<br>2. The coarse-grained MD simulations were performed using the GENESIS 1.7.1 (https://mdgenesis.org).<br>3. Atomic force microscopy (AFM) was performed using a Bruker Dimension Icon system.<br>4. Figures were created using Chimerax 1.8 (for snapshot of protein and RNA structures), Vmd 1.9.3 (for volmap of protein and RNA), PyMol 2.5.0 (for movies), official Basler software (for images of droplets), Nanoscope (for AFM imaging) and Python 3.10.12 (for graphs).
Data analysis	We used the following programs for data analysis:<br>1. Python 3.10.12 (https://www.python.org).<br>2. Vmd1.9.3 (https://www.ks.uiuc.edu).<br>3. Basler (https://www.baslerweb.com/).<br>4. Nanoscope (https://www.bruker.com/).

For manuscripts utilizing custom algorithms or software that are central to the research but not yet described in published literature, software must be made available to editors and reviewers. We strongly encourage code deposition in a community repository (e.g. GitHub). See the Nature Portfolio guidelines for submitting code & software for further information.

Data

Policy information about availability of data

All manuscripts must include a data availability statement. This statement should provide the following information, where applicable:

- Accession codes, unique identifiers, or web links for publicly available datasets
- A description of any restrictions on data availability
- For clinical datasets or third party data, please ensure that the statement adheres to our policy

The source data supporting the graphs and input files for MD simulations are available via the Figshare database (https://doi.org/10.6084/m9.figshare.29605127) and the institutional repository (https://box.nju.edu.cn/d/eb918ffb092246cf8971/).

Research involving human participants, their data, or biological material

Policy information about studies with human participants or human data. See also policy information about sex, gender (identity/presentation), and sexual orientation and race, ethnicity and racism.

Reporting on sex and gender	N/A
Reporting on race, ethnicity, or other socially relevant groupings	N/A
Population characteristics	N/A
Recruitment	N/A
Ethics oversight	N/A

Note that full information on the approval of the study protocol must also be provided in the manuscript.

Field-specific reporting

Please select the one below that is the best fit for your research. If you are not sure, read the appropriate sections before making your selection.

☒ Life sciences ☐ Behavioural & social sciences ☐ Ecological, evolutionary & environmental sciences

For a reference copy of the document with all sections, see nature.com/documents/nr-reporting-summary-flat.pdf

Life sciences study design

All studies must disclose on these points even when the disclosure is negative.

Sample size	In all-atom molecular dynamics (MD) simulations, the system comprised millions of atoms simulated for 100 ns. Within this time, the co-condensation of the CTDs and pgRNA was sufficiently observed. For coarse-grained MD simulations, we performed three independent 10^8-step runs per system, each initiated with a distinct random seed. Sample sizes were chosen to ensure efficient sampling of pgRNA phase separation events. Biochemical experiments, replicated three times, confirmed that RNA molecules undergo phase separation regulated by the HBV core protein CTD peptide, demonstrating reliable consistency between computational and experimental results. Atomic force microscopy (AFM) experiments were repeated two times to confirm the occurrence of phase separation on the CTD-surface.
Data exclusions	No data were excluded from analysis.
Replication	In coarse-grained MD simulations, we performed three independent runs per system configuration. All simulations consistently revealed successful replication of the phase behavior. For biochemical and AFM experiments, we repeated the phase behavior studies three times, with each demonstrated the co-condensation of the HBV core protein's CTD and RNA molecules.
Randomization	For simulations, multiple independent runs were performed using distinct random seeds. Temperature control was maintained in all simulations via Langevin dynamics. For experiments, replicates of biochemical and AFM assays were prepared from independently mixed reaction solutions.
Blinding	The simulations and experimental samples are randomized appropriately, and all conditions are identical except for the variables under comparison; therefore, blinding procedures are not applicable.

Reporting for specific materials, systems and methods

We require information from authors about some types of materials, experimental systems and methods used in many studies. Here, indicate whether each material, system or method listed is relevant to your study. If you are not sure if a list item applies to your research, read the appropriate section before selecting a response.

Materials & experimental systems

n/a	Involved in the study
☒	☐ Antibodies
☒	☐ Eukaryotic cell lines
☒	☐ Palaeontology and archaeology
☒	☐ Animals and other organisms
☒	☐ Clinical data
☒	☐ Dual use research of concern
☒	☐ Plants

Methods

n/a	Involved in the study
☒	☐ ChIP-seq
☒	☐ Flow cytometry
☒	☐ MRI-based neuroimaging

Plants

Seed stocks	N/A
Novel plant genotypes	N/A
Authentication	N/A
